# Circulating tumor DNA as prognostic markers of relapsed breast cancer: a systematic review and meta-analysis

**DOI:** 10.1016/j.jncc.2024.01.003

**Published:** 2024-01-23

**Authors:** Na'na Guo, Qingxin Zhou, Xiaowei Chen, Baoqi Zeng, Shanshan Wu, Hongmei Zeng, Feng Sun

**Affiliations:** 1 Hebei Province Centers for Disease Control and Prevention, Shijiazhuang, China; 2 Tianjin Centers for Disease Control and Prevention, Tianjin, China; 3 Department of Epidemiology and Biostatistics, School of Public Health, Peking University, Beijing, China; 4 Department of Science and Education, Peking University Binhai Hospital, Tianjin, China; 5 Clinical Epidemiology and EBM Unit, National Clinical Research Center for Digestive Diseases, Beijing Friendship Hospital, Capital Medical University, Beijing, China; 6 National Central Cancer Registry, National Cancer Center/National Clinical Research Center for Cancer/Cancer Hospital, Chinese Academy of Medical Sciences and Peking Union Medical College, Beijing, China; 7 Key Laboratory of Major Disease Epidemiology, Ministry of Education (Peking University), Beijing, China

**Keywords:** ctDNA, Breast cancer, Prognostic outcome prediction, Meta-analysis

## Abstract

**Objective:**

Circulating tumor DNA (ctDNA) is increasingly being used as a potential prognosis biomarker in patients of breast cancer. This review aims to assess the clinical value of ctDNA in outcome prediction in breast cancer patients throughout the whole treatment cycle.

**Methods:**

PubMed, Web of Science, Embase, Cochrane Library, Scopus, and clinical trials.gov were searched from January 2016 to May 2022. Conference abstracts published in last three years were also included. The following search terms were used: ctDNA OR circulating tumor DNA AND breast cancer OR breast carcinoma. Only studies written in English languages were included. The following pre-specified criteria should be met for inclusion: (1) observational studies (prospective or retrospective), randomized control trials, case-control studies and case series studies; (2) patients with breast cancer; (3) ctDNA measurement; (4) clinical outcome data such as objective response rate (ORR), pathological complete response (pCR), relapse-free survival (RFS), overall survival (OS), and so on. The random-effect model was preferred considering the potential heterogeneity across studies. The primary outcomes included postoperative short-term outcomes (ORR and pCR) and postoperative long-term outcomes (RFS, OS, and relapse). Secondary outcomes focused on ctDNA detection rate.

**Results:**

A total of 30 studies, comprising of 19 cohort studies, 2 case-control studies and 9 case series studies were included. The baseline ctDNA was significantly negatively associated with ORR outcome (Relative Risk [RR] = 0.65, 95% confidence interval [CI]: 0.50–0.83), with lower ORR in the ctDNA-positive group than ctDNA-negative group. ctDNA during neoadjuvant therapy (NAT) treatment was significantly associated with pCR outcomes (Odds Ratio [OR] = 0.15, 95% CI: 0.04–0.54). The strong association between ctDNA and RFS or relapse outcome was significant across the whole treatment period, especially after the surgery (RFS: Hazard Ratio [HR] = 6.74, 95% CI: 3.73–12.17; relapse outcome: RR = 7.11, 95% CI: 3.05–16.53), although there was heterogeneity in these results. Pre-operative and post-operative ctDNA measurements were significantly associated with OS outcomes (pre-operative: HR = 2.03, 95% CI: 1.12–3.70; post-operative: HR = 6.03, 95% CI: 1.31–27.78).

**Conclusions:**

In this review, ctDNA measurements at different timepoints are correlated with evaluation indexes at different periods after treatment. The ctDNA can be used as an early potential postoperative prognosis biomarker in breast cancer, and also as a reference index to evaluate the therapeutic effect at different stages.

## 1 Introduction

Breast cancer has overtaken lung cancer to become the most common cancer and the fifth leading cause of cancer death worldwide. In 2020, breast cancer accounted for approximately 24.5% of all cancer cases and 15.5% of cancer deaths in women.^1^ New cases of breast cancer are expected to reach 4.4 million in 2070.^2^

Although much progress has been made in the diagnosis and treatment,^3^ screening, and monitoring of breast cancer, there are still great limitations in predicting the prognosis of breast cancer, due to the invasive nature of existing diagnostic and detection methods. Commonly used follow-up biomarkers have shown varied degrees of limitations.

Local relapse is commonly diagnosed by X-ray, ultrasound, or biopsy. However, these methods have limitations: radiation exposure is harmful, biopsy is invasive, and whole-body computerized tomography is more expensive and less sensitive.^4^^,^^5^ For distal metastases, the common diagnosis is a serum tumor marker, which is an easily repeatable and relatively inexpensive tool, and the CEA-TPA-CA15.3 combination increases sensitivity in the "early" diagnosis of postoperative metastatic breast cancer, but decreases specificity.^6^^,^^7^

Based on the above, circulating tumor DNA (ctDNA) is more accurate because it can fully detect target genes in a single blood collection and analyze individual genetic variations in patients. ctDNA, which is shed into the blood by tumor and accounts for 0.01%–90% of the total circulating cell free DNA, is considered an important component of liquid biopsy.^8^ ctDNA can be detected via targeting tumor-specific mutations, structural variants, copy number alterations, and epigenetic features through polymerase chain reaction (PCR) or next generation sequencing (NGS) assays.^9^ ctDNA detection has been well-established and widely used in hematological cancers, but remains challenging in solid tumors.^10^ Studies suggest that evidence is accumulating between the use of ctDNA in solid tumors prognosis such as breast cancer, lung cancer, colorectal cancer, pancreatic cancer, etc.^11^, ^12^, ^13^, ^14^ Currently, reviews and meta-analysis of the early screening and prognostic value of ctDNA are mostly limited to studies of a certain stage or outcome measure in breast cancer.^15^, ^16^, ^17^, ^18^ For instance, Carolyn Cullinane et al. reported the association of ctDNA with disease-free survival (DFS) in BC, and Mikail et al. reported ctDNA for risk of recurrence assessment in patients.

In order to evaluate the application of ctDNA detection in predicting the risk of breast cancer recurrence and treatment effect, this study systematically searched domestic and foreign studies on the application of ctDNA detection in breast cancer, and effectively integrated them by meta-analysis, with a view to clarifying the association between ctDNA detection at different times and breast cancer outcomes.

## 2 Methods

### 2.1 Protocol and registration

This systematic review and meta-analysis was conducted according to the Preferred Reporting Items for Systematic Reviews and Meta Analyses (PRISMA) guidelines^19^ and Meta-analysis of Observational Studies in Epidemiology (MOOSE) to identify studies that assess the association of ctDNA and clinical outcomes in breast cancer patients. The study protocol had been prospectively registered on PROSPERO (CRD 42022331326).

### 2.2 Search strategy

The electronic databases PubMed, Web of Science, Embase, Cochrane Library, Scopus, and clinical trials.gov databases were searched from January 2016 to May 2022. The detailed search strategy is available in Supplementary materials. References of relevant sources were hand-searched for relevant studies, and surfaced one article that was not captured by the search strategy.

After removing the duplicates automatically (Endnote X8, Clarivate, Philadelphia, PA) and manually, the titles and abstracts were first screened and then the potential eligible articles were full-text reviewed, based on the eligibility criteria. This process was performed independently by two authors and any discrepancies were resolved by discussion (Q.X. Zhou and X.W. Chen).

### 2.3 Study selection

The following prespecified inclusion criteria included: i) original articles encompassing observational studies (prospective or retrospective), randomized control trials, cross-sectional studies or case series studies; ii) studies including patients with breast cancer in perioperative period; iii) documented collection and measurement of ctDNA. All methods of ctDNA detection and analysis were allowed, presented as a binary classification (positive/negative), given the lack of a gold standard and of direct comparisons between the various methods; iv) clinical outcome data, such as DFS, relapse-free survival (RFS), overall survival (OS), objective response rate (ORR) or pathological complete response (pCR); v) written in the English language; vi) both full-length articles and conference abstracts were included. Full-length articles were peer-reviewed.

Exclusion criteria: i) studies with no primary data (review articles, editorials, comments), studies with a sample size of 5 or less, or ongoing studies without results; ii) only the elevated and reduced ctDNA levels or cell-free DNA was measured; iii) other cancers or diseases; and iv) studies focusing on diagnosis or screening outcomes. Besides, studies reporting on similar cohorts within the same time period were also assessed. The most up-to-date and largest study was chosen.

### 2.4 Data extraction and synthesis

For the purpose of this analysis, ctDNA was considered a binary variable (positive vs negative). The following variables were extracted from the selected literature, i) general information: title, first author, year, journal, design, country; ii) basic population characteristics: sample size, cancer stage, cancer grade, follow-up duration; iii) ctDNA analysis information: measurement methods and timepoints, type of assay, positive definition; iv) outcome measurements: RFS (composite endpoints including RFS, event-free survival (EFS) , Disease Free Survival (DFS) , and Distant disease free survival (DDFS) , depending on the study design), pCR, OS, relapse rate and ctDNA detection rate.

### 2.5 Outcomes and measures

The primary endpoint of the meta-analysis included: (1) postoperative short-term outcomes: ORR and pCR; (2) postoperative long-term outcomes: RFS, OS, and relapse. Secondary endpoints were (1) the ctDNA detection rate at different timepoints; (2) the negative conversion rate of ctDNA at different times.

### 2.6 Quality assessment

The Newcastle-Ottawa Scale (NOS) for observational studies (cohort study and case-control study) and the NICE (National Institute for Health and Care Excellence) quality assessment tool for case series studies were used.

### 2.7 Statistical analysis

The meta-analyses were conducted separately for each timepoint, including baseline (before any treatment), during neoadjuvant therapy (NAT), before surgery (after NAT), and after surgery. To summarize the overall effect, the hazard ratio (HR) with 95% confidence intervals (CIs) was calculated for the RFS and OS analysis, the risk ratio (RR) and odds ratio (OR) with 95% CI was calculated for the relapse and pCR analysis, respectively. Heterogeneity was assessed and reported using *I^2^* statistics (greater than 50% considered as significant heterogeneity) and Cochran's Q test. Fixed-effect model was fitted when there was no significant heterogeneity (*I^2^* ≤ 50%); vice versa when there was significant heterogeneity (*I^2^* > 50%), a random-effect model was preferred. All reported *P* values were two-sided, and *P* < 0.05 was considered statistically significant. The Q test was used to detect the difference among the different ctDNA measurement timepoints subgroups. For the meta-analysis of ctDNA detection rates, the pooled estimates and corresponding 95% CI were calculated. To handle extreme proportions, the Freeman-Tukey double arcsine transformation was chosen and the random-effect model was fitted. Funnel plot analysis and Egger's test were performed to detect publication bias. All analyses were performed using R statistical software, version 4.0.0 (R packages *metafor* and *meta*). Sensitivity analysis was performed with the leave-one-out methods or removed the abstract outcomes with the only published literature pooled to measure the stability of pooled results when data available.

## 3 Results

### 3.1 Risk of bias

Risk assessment of bias was performed according to study type (total score: 8). Nineteen cohort studies had risk bias scores of 6–8 (Supplementary Table 5), two case-control studies had risk bias scores of 4–5 (Supplementary Table 6), and nine case-series studies had risk bias scores of 3–5 (Supplementary Table 7).

### 3.2 Literature search results

**Fig. 1:**
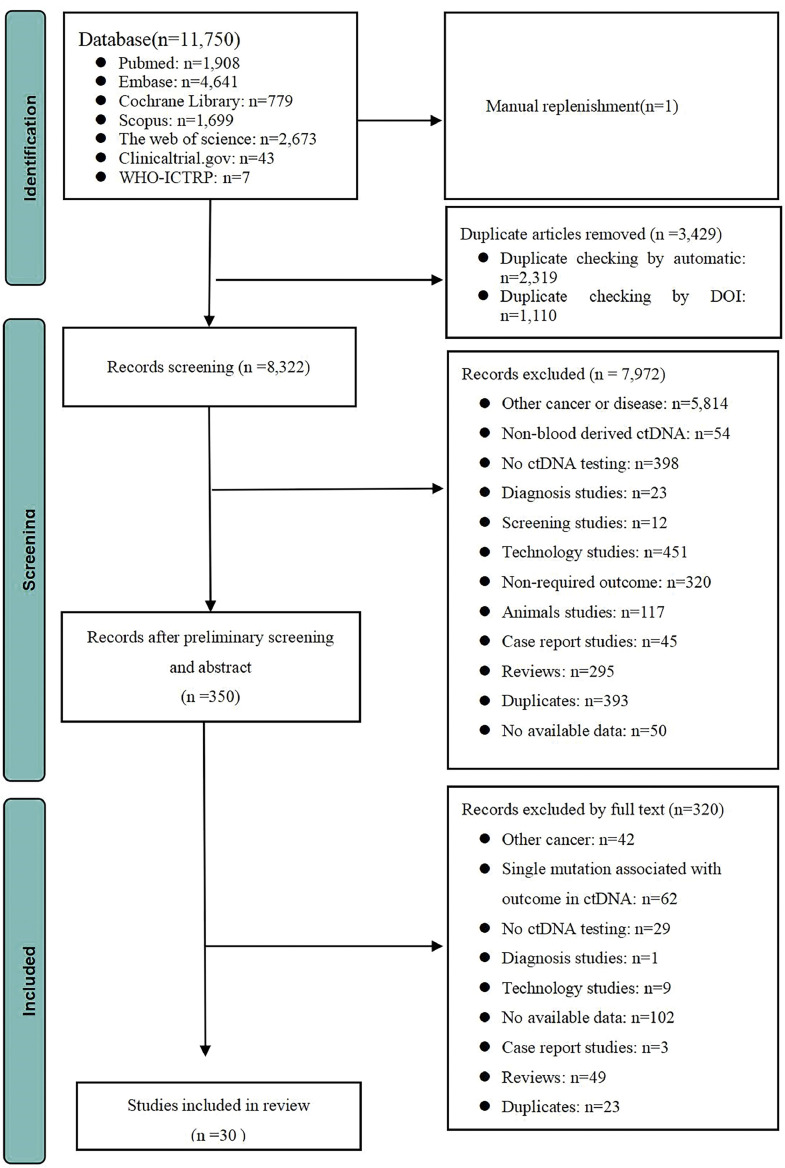
Flowchart of records inclusion and exclusion.

A total of 30 records were included, including 18 published articles and 12 conference abstracts. The screening process is shown in Fig. 1. The publication year ranged from 2016 to 2022, and 28 articles reported the country of the study population: 6 articles from China, 7 articles from the United States, 4 from the United Kingdom, 3 from Japan, 2 each from France and Belgium, and 1 article each from Australia, Germany, Canada and Italy (Table 1).^20^, ^21^, ^22^, ^23^, ^24^, ^25^, ^26^, ^27^, ^28^, ^29^, ^30^, ^31^, ^32^, ^33^, ^34^, ^35^, ^36^, ^37^, ^38^, ^39^, ^40^, ^41^, ^42^, ^43^, ^44^, ^45^, ^46^, ^47^, ^48^, ^49^ According to the timing of ctDNA measurements, 14 studies reported baseline, 5 studies reported during NAT, and 9 studies reported after NAT and before surgery. Two studies reported before surgery (with or without NAT), and 15 studies reported after surgery (Supplementary Table 1 in). By reported outcomes, 13 studies reported RFS outcomes, 6 reported OS outcomes, 10 reported relapse outcomes, 8 reported pCR outcomes, 2 reported ORR outcomes, 27 reported ctDNA positive detection rates, and 6 reported rates of ctDNA turnover (Supplementary Table 1 in). Twenty-four studies reported clinical follow-up periods ranging from 12 months to 4.8 years (Supplementary Table 2).

**Table 1:** Basic information of the included records.

Author	Publication type	Registration No.	Sample, n	Country	Cohort Time	Multi center	Prospective	Age, median, years	Treatment
L Cavallone,^20^	Article	NCT01276899 Q-CROC-03 trial	26	Canada and United States	08/2010–12/2013	Y	–	48.9	Anthracycline/taxane or taxane-alone; Surgery
Y.H Chen,^21^	Article	NCT01074970 BRE09–146	38	United States	03/2010–05/2013	Y	Y	47 (range: 21–66)	Anthracycline or cyclophosphamide or taxane or carboplatin; Surgery
I Garcia-Murillas,^22^	Article	The ChemoNEAR or the Plasma DNA study	170	United Kingdom	11/2011–10/2016	Y	Y	54±11	Surgery
S Li,^23^	Article	NCT03260192	44	China	2013–2015	N	Y	45 (range:26–68)	Doxorubicin or epirubicin or cyclophosphamide or docetaxel or herceptin; Surgery
M.J.M Magbanua,^24^	Article	NCT01042379 I-SPY 2 TRIAL	84	United States	–	Y	N	ctDNA^+^: mean 45.7 ctDNA^-^: mean 50.6	Paclitaxel+anthracycline; Surgery
E Ortolan,^25^	Article	–	31	France	04/2013–02/2017	–	Y	< 50:19 ≥ 50:12	Anthracycline/taxane or anthracycline/taxane plus platins; Surgery
M Radovich,^26^	Article	NCT02101385 BRE12-158	142	United States	03/2014- 12/2018	Y	–	≤ 45: 46; 46–60: 64 61–75: 31; ≥ 76: 1	Anthracycline; Surgery
R.C Coombes,^27^	Article	EBLIS	49	United Kingdom	–	Y	Y	57 (range: 38–81)	Anthracycline/taxane; Surgery
F Riva,^28^	Article	NCT02220556 CTC-CEC DNA study	36	France	01/2013–05/2014	–	Y	< 50:19 ≥ 50:17	5-fluorouracil, epirubicin and cyclophosphamide or cyclophosphamide–anthracycline; Surgery
F Rothe,^29^	Article	NeoALTTO	69	Belgium	01/2008–05/2010	Y	–	51 (range: 23–80)	Anti-HER2 therapies+paclitaxel; Surgery
H Takahashi,^30^	Article	–	87	Japan	07/2012–08/2015	–	Y	–	Paclitaxel+5-fluorouracil+epirubicin+cyclophosphamide; Surgery
Y Chen,^31^	Article	–	80	China	01/2017–01/2019	N	–	61.28±13.57	Exemestane+neoadjuvant endocrine therapy; Surgery
P.H Lin,^32^	Article	–	95	China	–	–	–	50±8.8	NAT; Surgery
T Yoshinami,^33^	Article	–	62	Japan	2007–2012	N	–	ctDNA^+^: 55 (range 35–80) ctDNA^-^: 51 (range 36–82)	Without preoperative systemic therapies; Surgery
M Lipsyc-Sharf,^34^	Article	–	83	United States	03/2018–12/2020	N	Y	53 (range 29–71)	NAT; Surgery
Q Zhou,^35^	Article	ABCSG-34 trial	142	Austria	–	Y	Y	≤ 55:55 > 55:87	Anthracycline/cyclophosphamide followed by taxane or endocrine therapy alone or combination with the therapeutic cancer vaccine tecemotide; Surgery
S.D Cosimo,^36^	Conference abstract	–	27	Italy	–	–	–	–	Surgery
W Janni,^37^	Conference abstract	The BRandO BiO registry study	38	Germany	–	–	–	–	Surgery
E Agostinetto,^38^	Conference abstract	–	38	Belgium	–	N	–	–	NAT; Surgery
P Sharma,^39^	Conference abstract	–	–	United States	2011–2018	–	–	47	NAT; Surgery
M.J.M Magbanua,^40^	Conference abstract	I-SPY 2 TRIAL	132	United States	–	–	–	–	Paclitaxel+standard adjuvant therapy
J Lan,^41^	Article	–	20	China	–	N	–	42 (range:33–79)	Surgery
X Zhang,^42^	Article	–	102	China	–	N	–	51.06±7.87	Surgery
D.M Carraro,^43^	Conference abstract	–	16	–	–	–	–	–	NAT
N Turner,^44^	Conference abstract	c-TRAK TN trial	161	United Kingdom	01/2018–12/2019	Y	Y	–	NAT
Y Takahashi,^45^	Conference abstract	–	86	Japan	04/2018–04/2019	–	–	–	–
R.J Cutts,^46^	Conference abstract	ChemoNEAR	22	United Kingdom	–	–	N	–	Surgery
F Lynce,^47^	Conference abstract	OXEL	33	United States	08/2018–06/2021	–	–	51±12	Anthracycline or a taxane
F Ma,^48^	Conference abstract	NCT02041338	31	China	01/2014–06/2017	N	Y	–	Paclitaxel+carboplatin or paclitaxel or paclitaxel+epirubicin
M Fedyanin,^49^	Conference abstract	–	66	–	–	N	Y	–	Surgery

Abbreviations*:* -, not reported; N, no; NAT, neoadjuvant therapy; Y, yes.

### 3.3 ctDNA and pCR

**Fig. 2:**
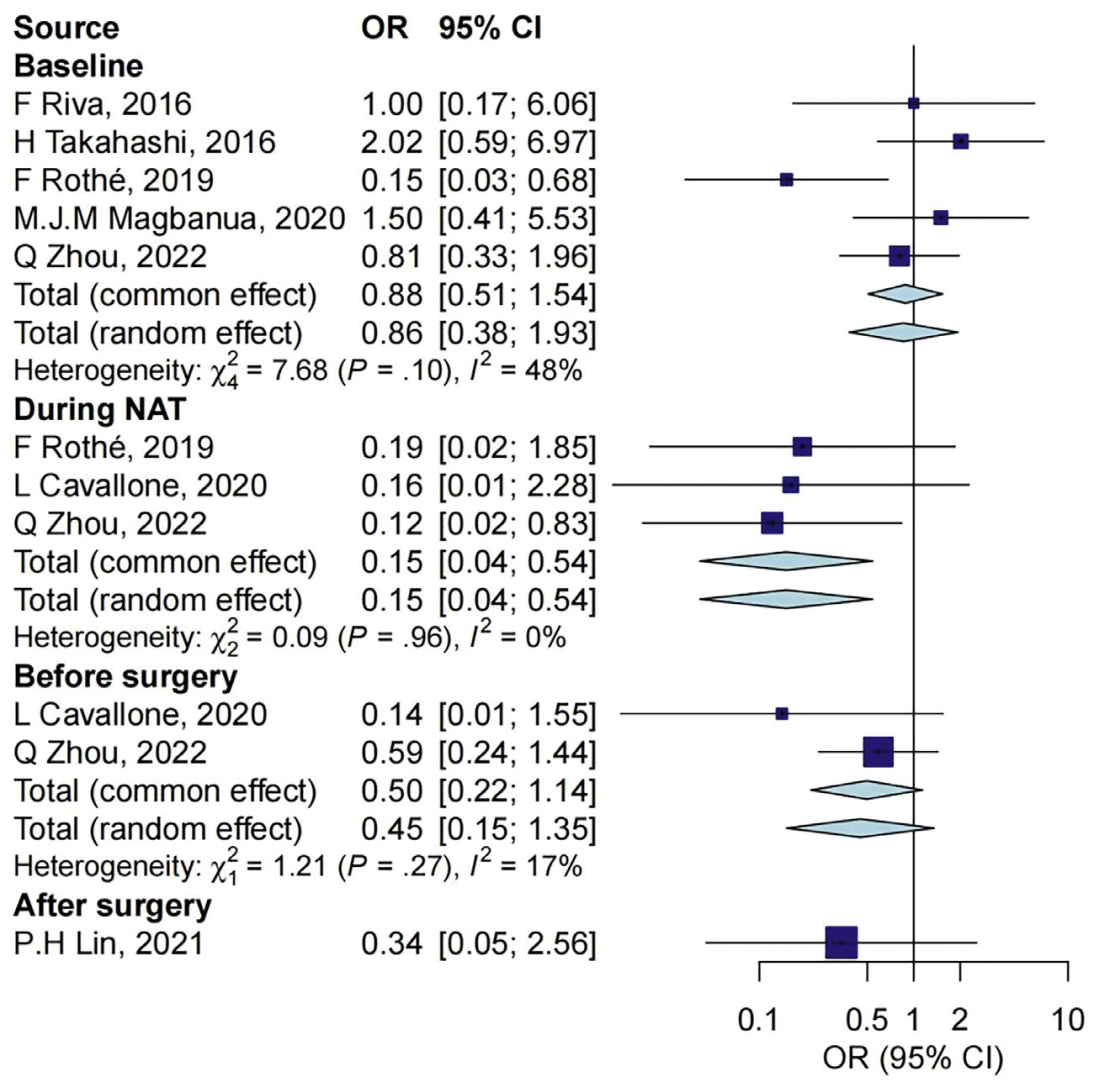
Association between ctDNA measurements and pCR outcome at different timepoints. CI, confidence interval; NAT, neoadjuvant treatment; OR, odds ratio.

In the 8 studies that reported ctDNA and pCR, there were 5 that reported the relationship between ctDNA measurement at baseline and pCR with 414 samples, 3 reported the relationship during NAT with 206 samples, 2 reported the relationship before surgery with 201 samples, and 1 reported the relationship after surgery with 95 samples (Fig. 2). Compared with individuals with negative ctDNA, those with positive ctDNA during NAT treatment had a significantly lower rate of pCR (OR = 0.15, 95% CI: 0.04–0.54), while no significant associations were detected in other three periods.

### 3.4 ctDNA and ORR

**Fig. 3:**
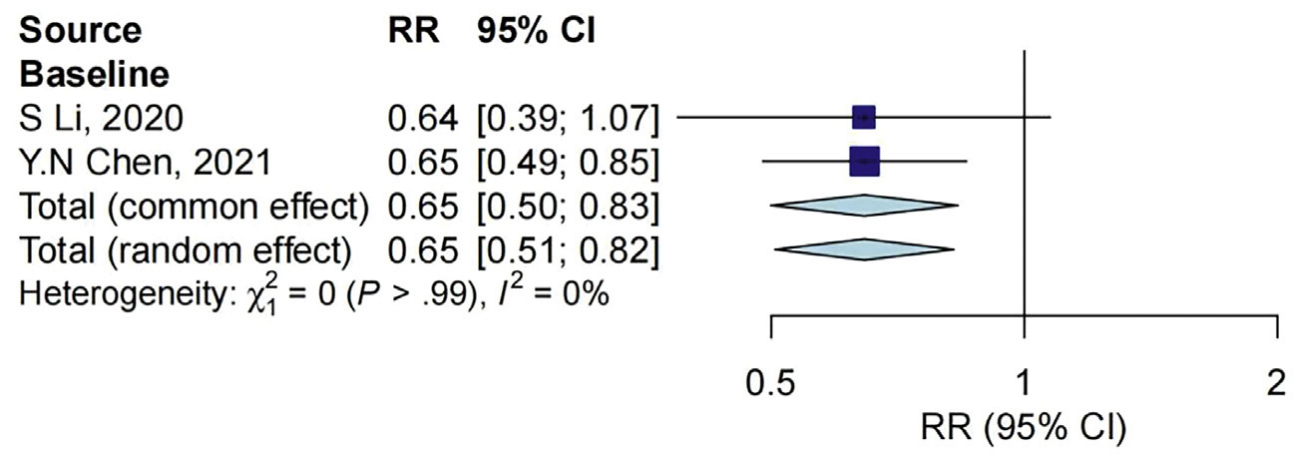
Association between ctDNA measurements and ORR outcome at different timepoints. CI, confidence interval; RR, relative risk.

There are only 2 studies that reported ctDNA and ORR, at baseline with 124 samples. Results showed that the ORR rate was lower in the ctDNA-positive group than in the ctDNA-negative group (RR = 0.65, 95% CI: 0.50–0.83) (Fig. 3).

### 3.5 ctDNA and RFS

**Fig. 4:**
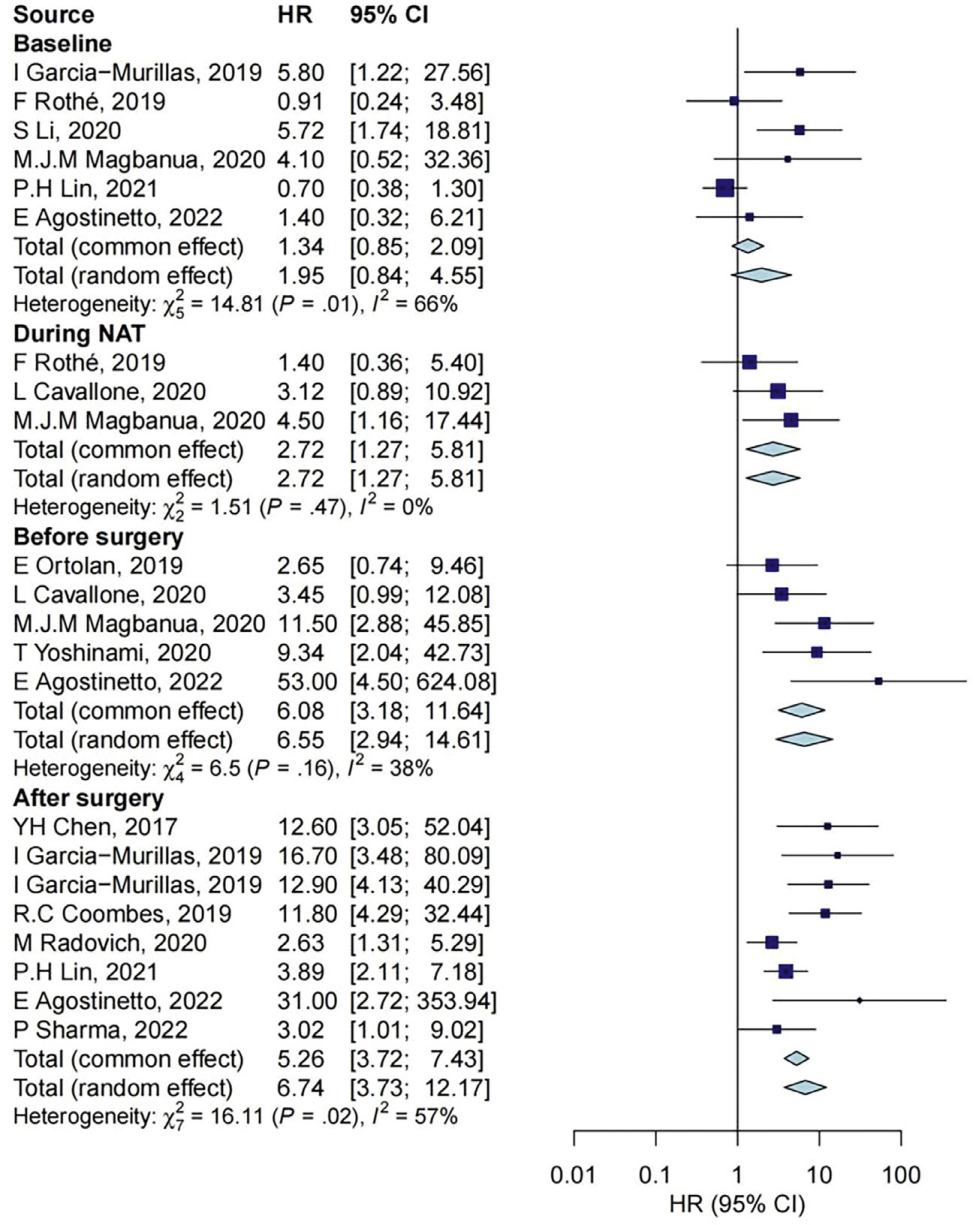
Association between ctDNA measurements and RFS outcome at different timepoints. CI, confidence interval; HR, hazard ratio; NAT, neoadjuvant treatment.

In the 13 studies that reported ctDNA and RFS, ctDNA measurements were reported at baseline for 6 studies with 401 samples, during NAT treatment for 3 studies with 159 samples, before surgery for 5 studies with 209 samples, and after surgery for 8 studies with 548 samples. The RFS of individuals with positive ctDNA was similar to that with negative ctDNA at baseline measurements (HR = 1.95, 95% CI: 0.84–4.55). At the other three timepoints, compared with individuals with negative ctDNA, those with positive ctDNA during NAT treatment, before surgery, and after surgery had significantly shorter RFS (HR = 2.72, 95% CI: 1.27–5.81; HR = 6.08, 95% CI: 3.18–11.64; HR = 6.74, 95% CI: 3.73–12.17; respectively). The effect size of the association between postoperative ctDNA sampling and outcome was higher than that at other timepoints, as shown in Fig. 4. Further comparisons were made between groups, baseline vs during NAT (*P* = 0.570), baseline vs before surgery (*P* = 0.042), and baseline vs after surgery (*P* = 0.019).

### 3.6 ctDNA and relapse outcome

**Fig. 5:**
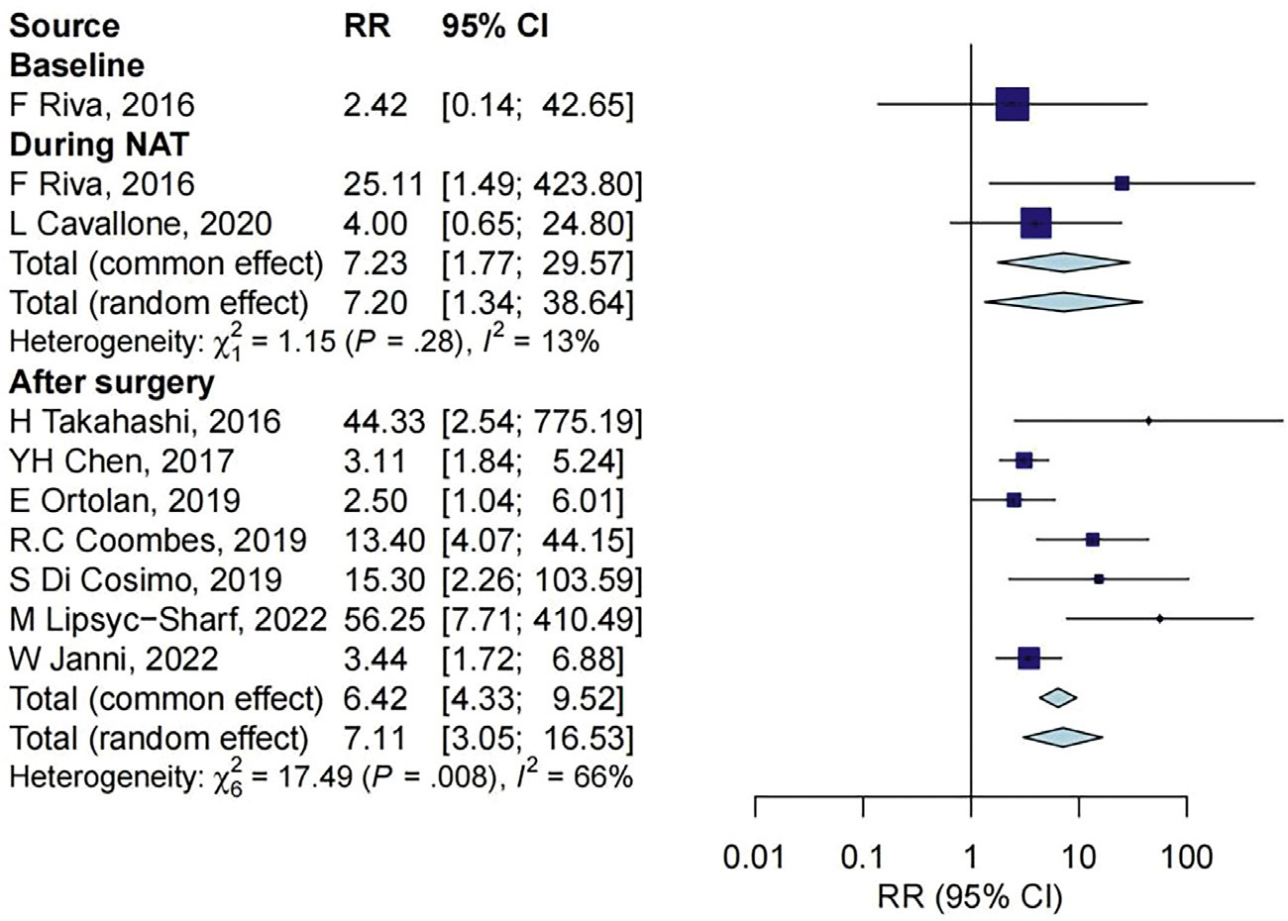
Association between ctDNA measurements and relapse outcome at different timepoints. CI, confidence interval; NAT, neoadjuvant treatment; RR, relative risk.

In the 11 studies that reported ctDNA and the rate of relapse, ctDNA measurements were reported at baseline for 1 study with 36 samples, during NAT treatment for 2 studies with 56 samples, before surgery for 3 studies with 105 samples, and after surgery for 7 studies with 308 samples, as shown in Fig. 5. The results showed that the rate of relapse in individuals with negative ctDNA was similar to those with positive ctDNA at baseline (RR = 2.42, 95% CI: 0.14–42.65). At the other three timepoints, compared with individuals with negative ctDNA, those with positive ctDNA during NAT treatment, before surgery, and after surgery had significantly higher rates of relapse (RR = 7.23, 95% CI: 1.77–29.57; RR = 3.76, 95% CI: 1.65–8.57; RR = 7.11, 95% CI: 3.05–16.53; respectively).

The effect sizes of ctDNA associated with outcome at four timepoints were compared, and there was no statistically significant difference in the effect size at different timepoints (*P* = 0.736).

### 3.7 ctDNA and OS

**Fig. 6:**
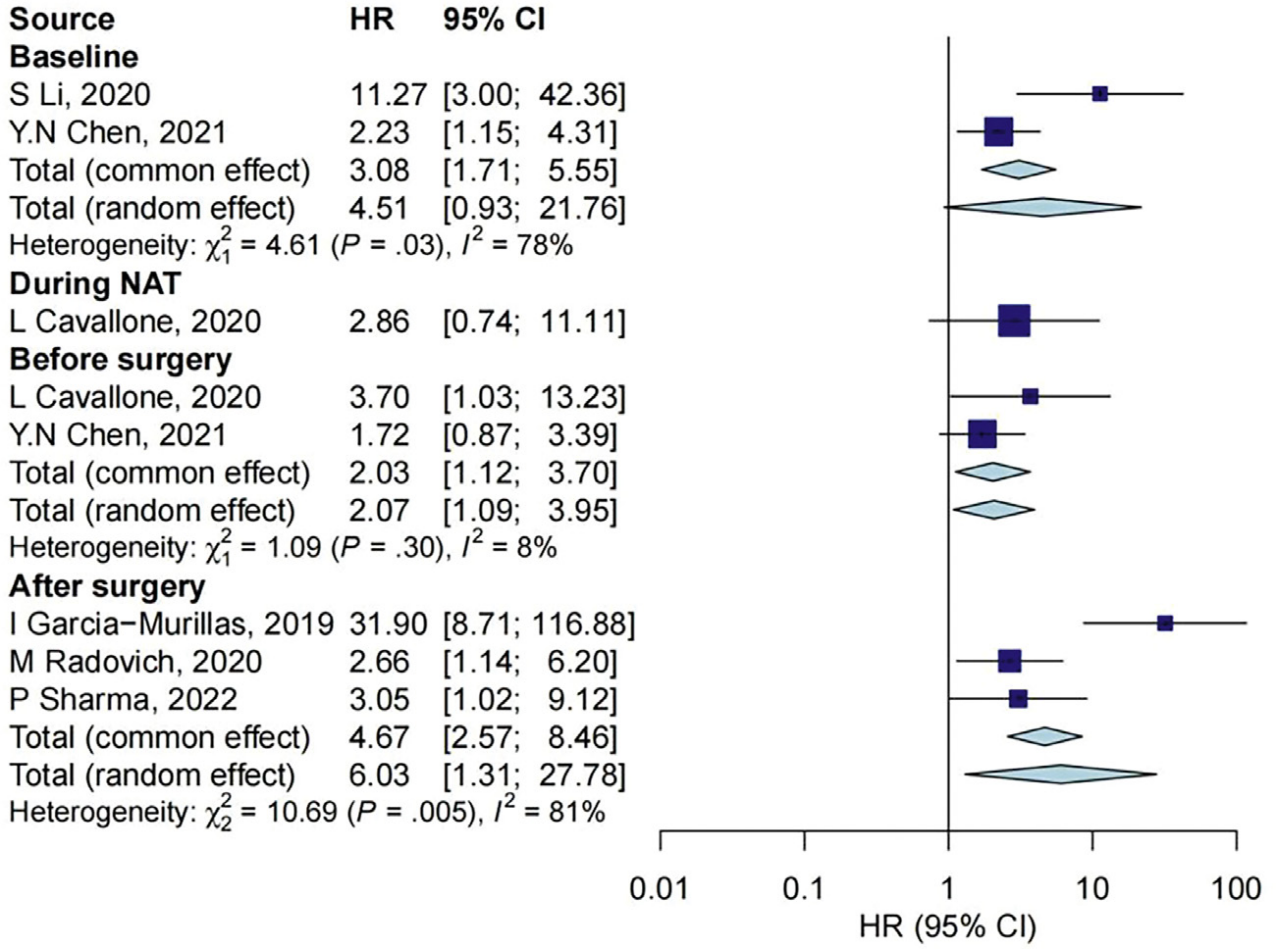
Association between ctDNA measurements and OS outcome at different timepoints. CI, confidence interval; HR, hazard ratio; NAT, neoadjuvant treatment.

In the 6 studies that reported ctDNA and OS, ctDNA measurements were reported at baseline for 2 studies with 124 samples, during NAT treatment for 1 study with 21 samples, before surgery for 2 studies with 103 samples, after surgery for 3 studies with 232 samples, as shown in Fig. 6. The OS in individuals with negative ctDNA was similar to those with positive ctDNA at baseline and during NAT treatment (HR = 4.51, 95% CI: 0.93–21.76; HR = 2.86, 95% CI: 0.74–11.11; respectively). For the ctDNA results before and after surgery, the OS in individuals with negative ctDNA was significantly longer than those with positive ctDNA (HR = 2.03, 95% CI: 1.12–3.70; HR = 6.03, 95% CI: 1.31–27.78; respectively).

The effect values of ctDNA associated with outcome at four timepoints were compared, and there was no statistically significant difference in the effect values at different timepoints (*P* = 0.547).

### 3.8 The detection and turning rates of ctDNA

**Fig. 7:**
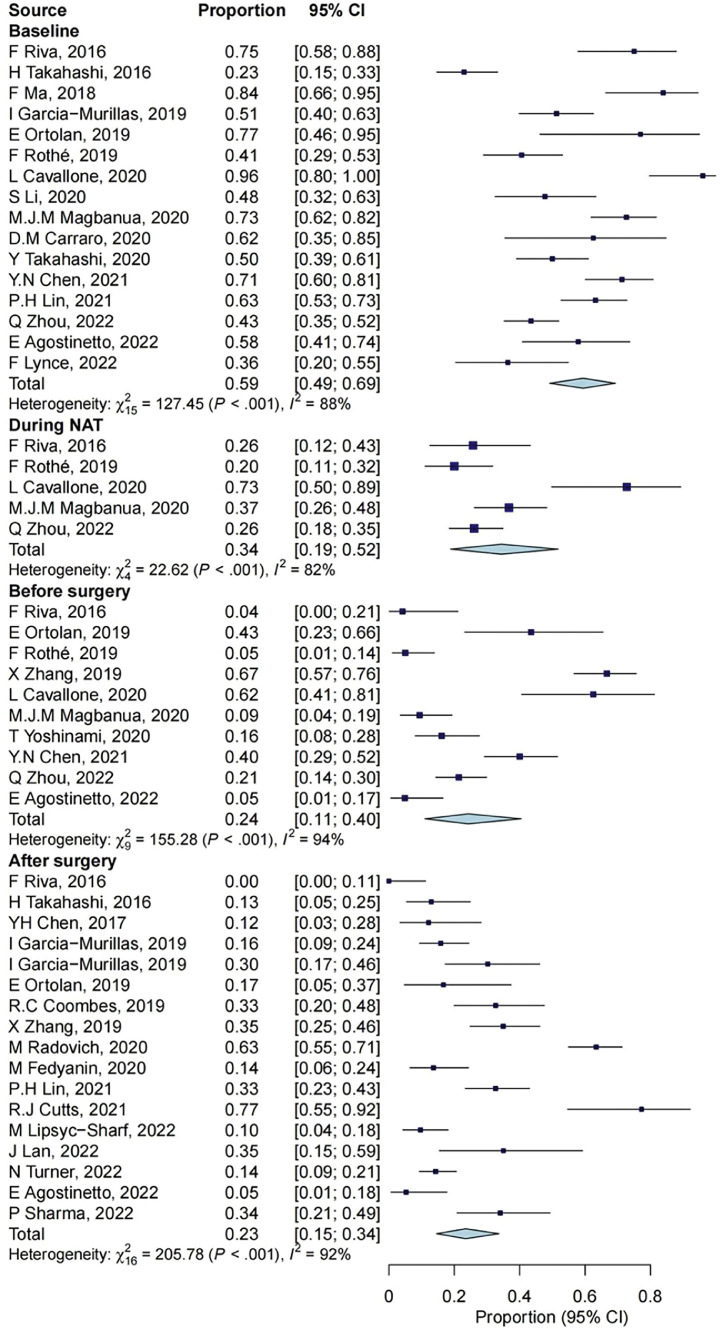
ctDNA detection rates at different timepoints. CI, confidence interval; NAT, neoadjuvant treatment.

Twenty-seven studies reported the ctDNA detection rates at different times (Supplementary Table 3). The detection rate of ctDNA at different timepoints showed a gradually decreasing trend (*P* < 0.001). The combined effect value of the two adjacent periods was significantly higher at baseline than in the NAT period (*P* = 0.015), and there was no statistical difference between the other two groups (vs before NAT: *P* = 0.381, vs after NAT: *P* = 0.929), as shown in Fig. 7.

**Fig. 8:**
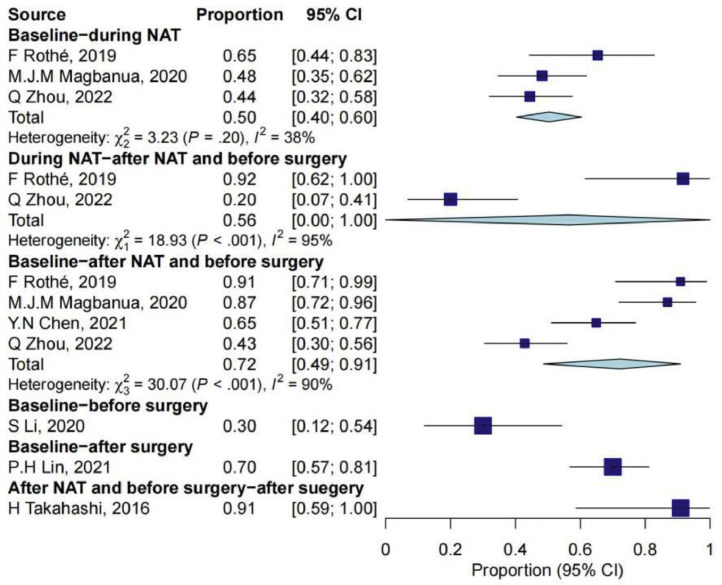
Negative conversion rate in ctDNA-positive patients of different periods. CI, confidence interval; NAT, neoadjuvant treatment.

Seven studies reported the negative conversion rate of ctDNA at different times. Three studies reported a negative ctDNA conversion rate of 49.66% (95% CI: 41.41%–57.92%) during NAT treatment in people with positive ctDNA (ctDNA+) at baseline. Two studies reported that for people with ctDNA+ during NAT treatment, the ctDNA conversion rate before surgery was 56.44% (95% CI: 0.00%–100.00%). One study reported that for people with ctDNA+ before surgery, the rate of ctDNA turning negative after surgery was 90.91% (95% CI: 64.99%–100.00%) (Supplementary Table 4 and Fig. 8).

### 3.9 Sensitivity analysis and publication bias

Sensitivity analysis was carried out using the method of document removal one by one, the method of removing conference abstracts, and the method of replacing single factor results with the results of multi-factor analysis. For the relationship between ctDNA results before surgery and RFS, the included literature was excluded one by one for sensitivity analysis, and there was no significant change in the study results. Some studies also reported the correlation between ctDNA and RFS after correcting for other influencing factors. After replacing the results of single factor analysis with the results of multi-factor analysis, the combined results were still significant (HR = 6.64, 95% CI: 2.46–17.89). The results showed that there was no significant change in the research results after sensitivity analysis, indicating that the results of different periods and different outcomes were stable (Supplementary Table 8).

## 4 Discussion

In this systematic review, 30 articles were eligible for meta-analysis and showed that positive ctDNA detection during the cycle of breast cancer treatment is associated with poorer prognosis. To the best of our knowledge, this is the first study that represents a comprehensive and pioneering effort to encompass all disease stages, full-cycle ctDNA detection, and multiple outcomes simultaneously.

### 4.1 ctDNA can make up for the inadequacy of traditional clinical diagnostic tools

Although precision medicine for breast cancer has made great progress in recent years, there are still many problems, such as tumor heterogeneity, cost of biopsy, and technical difficulties in molecular detection.^50^ Additionally, ctDNA can quickly and conveniently obtain continuous and balanced tumor gene information and analyze its expression differences.^51^, ^52^, ^53^ This study shows that ctDNA is closely related to clinical prognosis. Moreover, ctDNA detection is highly specific and more sensitive than imaging, and at least 7–13.5 months earlier than clinical recurrence.^54^, ^55^, ^56^, ^57^, ^58^ Therefore, ctDNA can make up for the deficiency of traditional clinical diagnostic tools and help medical personnel to identify the prognosis early and choose the appropriate clinical treatment plan.

### 4.2 ctDNA for breast cancer prognosis

NAT can reduce tumor volume and stage, and has become one of the important methods for clinical treatment of breast cancer.^59^ Postoperative pathological examination is the final means to evaluate the efficacy of NAT, and the prognosis of patients with pCR is significantly better than that of non-pCR patients.^60^ This study shows that the pCR of ctDNA-positive people during NAT is significantly lower than that of ctDNA-negative people. Therefore, ctDNA can make up for the deficiency of traditional clinical diagnostic tools and help medical personnel in complementing the use of serum tumor markers (CEA, CA15.3, and TPA) in the post-operative monitoring of breast cancer patients.

Positive ctDNA at baseline and during NAT was associated with early postoperative outcomes (pCR and ORR), while positive ctDNA before and after surgery was more likely to reflect long-term outcomes, such as RFS, OS, etc. Perhaps "positive ctDNA before and after surgery" has the potential to replace or refine more common prognostic markers as tissue biomarkers and genetic signatures. These studies suggest that ctDNA may become an important biological method for clinical assessment of breast cancer prognosis in the future.

### 4.3 Strengths and limitations

This review possesses many strengths. Firstly, it examined the associations of ctDNA with prognostic outcomes at multiple timepoints of treatment rather than a single timepoint. Secondly, sensitivity analyses after excluding the abstracts and using the results from the multivariate rather than the univariant model to confirm the stability of results were conducted, which is rare in other papers.

Certainly, this review inevitably has some limitations. First, there is some heterogeneity between the included studies, as there was no clinical gold standard or practical consensus. Outcome follow-ups and definitions varied, and we used some alternative outcomes, which may not include non-disease related deaths and may introduce confounders. Secondly, only studies that reported qualitative testing of ctDNA were included. The extent to which changes in quantitative ctDNA levels may impact outcomes remains unclear. In addition, in the process of systematic review, there were some problems in the limited number of literature and the small sample sizes, and thus the creditability of our results need to be verified by further large-scale studies. At the same time, due to the different definitions of ctDNA+ among different studies, the outcome measurement methods and population characteristics are different, so the heterogeneity of some effect sizes is large, which may affect the combined results.

## 5 Conclusions

ctDNA can be used as an early potential postoperative prognosis biomarker in breast cancer, and also as a reference index to evaluate the therapeutic effect at different stages. ORR and pCR as evaluation indexes in the first postoperative stage were associated with the positive rate of early baseline ctDNA and ctDNA during NAT. RFS and relapse as evaluation indexes in the second postoperative stage were correlated with the positive rate of ctDNA during NAT, before and after surgery. OS as the third stage of a postoperative effect evaluation index was related to the positive rate of ctDNA before and after surgery. In different periods (baseline, during NAT, before surgery, and after surgery), the positive detection rate of ctDNA gradually decreased, while the negative conversion rate gradually increased.

## Declaration of competing interest

The authors declare that they have no known competing financial interests or personal relationships that could have appeared to influence the work reported in this paper.

## Appendix Supplementary materials

Image, application 1
