# Supplementary material for: Circulating tumor DNA as prognostic markers of relapsed breast cancer: a systematic review and meta-analysis
Source: J Natl Cancer Cent. 2024 Jan 23;4(1):63–73. doi: 10.1016/j.jncc.2024.01.003 (PMC11256521; doi:10.1016/j.jncc.2024.01.003)
Supplement: Supplementary file 1 [file mmc1.pdf]

Supplementary materials

Circulating tumor DNA (ctDNA) as prognostic markers of relapsed breast cancer: a systematic review and meta-analysis

Na'na Guo, Qingxin Zhou, Xiaowei Chen, Baoqi Zeng, Shanshan Wu, Hongmei Zeng, Feng Sun

Literature search strategy and results (Search deadline: May 14, 2022)

1. Search strategy for PubMed

((((("neoplasms"[MeSH Terms] OR "breast cancer"[Title/Abstract] OR "lung cancer"[Title/Abstract] OR "colorectal cancer"[Title/Abstract] OR "colon cancer"[Title/Abstract] OR "rectal cancer"[Title/Abstract] OR "esophageal cancer"[Title/Abstract] OR "gastric cancer"[Title/Abstract] OR "liver cancer"[Title/Abstract] OR "hepatocellular carcinoma"[Title/Abstract] OR "pancreatic cancer"[Title/Abstract] OR "cholangiocarcinoma"[Title/Abstract] OR "gallbladder carcinoma"[Title/Abstract]) NOT ("hematologic neoplasms"[MeSH Major Topic] OR "neoplasms, plasma cell"[MeSH Major Topic] OR "multiple myeloma"[MeSH Major Topic] OR "leukemia"[MeSH Major Topic] OR "lymphoma"[MeSH Major Topic] OR "myelodysplastic syndromes"[MeSH Major Topic] OR "leukemia"[Title/Abstract] OR "myeloma"[Title/Abstract] OR "lymphoma"[Title/Abstract] OR "lymphoproliferative"[Title/Abstract] OR "myeloid"[Title/Abstract] OR "lymphoma*"[Title] OR "Hodgkin"[Title] OR "Immunoproliferative Small Intestinal"[Title/Abstract] OR "AML"[Title] OR "CLL"[Title] OR "CML"[Title] OR "APL"[Title] OR "HL"[Title] OR "NHL"[Title] OR "hematopoietic*"[Title] OR "haematologic*"[Title])) AND (("Circulating Tumor DNA"[MeSH Terms] OR "Circulating Tumor DNA"[Title/Abstract] OR "ctDNA"[Title/Abstract] OR "ct DNA"[Title/Abstract] OR ("molecular residual disease*"[Title/Abstract] OR "neoplasm, residual"[MeSH Major Topic] OR "minimal residual"[Title/Abstract] OR "MRD"[Title/Abstract] OR "residual minimal"[Title/Abstract] OR "measurable residual"[Title/Abstract])) NOT ("Chlamydia trachomatis"[MeSH Terms] OR "Chlamydia trachomatis"[Text Word] OR "calf thymus dna"[Supplementary Concept] OR "calf thymus"[Text Word])) AND ("Observational Study"[Publication Type] OR "Observational Studies as Topic"[MeSH Terms] OR "Cohort Studies"[MeSH Terms] OR "Case-Control Studies"[MeSH Terms] OR "Cross-Sectional Studies"[MeSH Terms] OR "observational stud*"[Title/Abstract] OR "Cohort"[Title/Abstract] OR "Follow-Up"[Title/Abstract] OR "longitudinal*"[Title/Abstract] OR "prospectiv*"[Title/Abstract] OR "retrospectiv*"[Title/Abstract] OR "Case-Control"[Title/Abstract] OR "Cross-Sectional"[Title/Abstract] OR "case series"[Title/Abstract] OR "single arm"[Title/Abstract] OR ("controlled clinical trial"[Publication Type] OR "Controlled Clinical Trials as Topic"[MeSH Terms] OR "Random Allocation"[MeSH Terms] OR "Double-Blind Method"[MeSH Terms] OR "single-blind method"[MeSH Terms] OR "Control Groups"[MeSH Terms] OR "cross-over studies"[MeSH Terms] OR "random*"[Title/Abstract] OR "placebo"[Title/Abstract] OR "trial"[Title/Abstract] OR "groups"[Title/Abstract] OR "crossover"[Title/Abstract] OR "cross-over"[Title/Abstract]))) NOT ("Animals"[MeSH Terms] NOT ("Humans"[MeSH Terms] AND "Animals"[MeSH Terms]))) NOT ("Review"[Title] OR "Review"[Title] OR "Meta"[Title] OR "Meta"[Title])) AND (2016:2022[pdat])

2. Search strategy for Embase

((('neoplasm'/exp OR 'breast cancer':ti,ab OR 'lung cancer':ti,ab OR 'colorectal cancer':ti,ab OR 'colon cancer':ti,ab OR 'rectal cancer':ti,ab OR 'esophageal cancer':ti,ab OR 'gastric cancer':ti,ab OR 'liver cancer':ti,ab OR 'hepatocellular carcinoma':ti,ab OR 'pancreatic cancer':ti,ab OR 'cholangiocarcinoma':ti,ab OR 'gallbladder carcinoma':ti,ab) NOT ('hematologic

disease'/exp OR 'multiple myeloma'/exp OR 'leukemia'/exp OR 'lymphoma'/exp OR 'leukemia':ti,ab OR 'myeloma':ti,ab OR 'lymphoma':ti,ab OR 'lymphoproliferative':ti,ab OR 'myeloid':ti OR 'myelodysplastic syndrome':ti,ab OR 'lymphoma*':ti OR 'hodgkin':ti,ab OR 'immunoproliferative small intestinal':ti,ab OR 'aml':ti OR 'cll':ti OR 'cml':ti OR 'apl':ti OR 'hl':ti OR 'nhl':ti OR 'hematopoietic*':ti OR 'haematologic*':ti) AND ('circulating tumor dna'/exp/mj OR 'circulating tumor dna':ab,ti OR 'ctdna':ab,ti OR 'ct dna':ab,ti OR 'minimal residual disease'/exp/mj OR 'molecular residual':ab,ti OR 'minimal residual':ab,ti OR 'mrd':ab,ti OR 'residual minimal':ab,ti OR 'measurable residual':ab,ti)) NOT ('chlamydiasis'/exp OR 'chlamydiasis':ab,ti OR 'chloroplast'/exp OR 'chloroplast':ab,ti OR 'chloroplast*':ab,ti OR 'etioplast*':ab,ti OR 'etioplast':ab,ti OR 'calf thymus':ab,ti) AND ([article]/lim OR [conference abstract]/lim OR [conference paper]/lim) AND [humans]/lim) NOT ('review':ti OR 'meta':ti) AND [2016-2022]/py AND [embase]/lim NOT [medline]/lim

3. Search strategy for Cochrane Library

#1	MeSH descriptor: [Neoplasms] explode all trees
#2	("breast cancer"):ti,ab,kw
#3	("lung cancer"):ti,ab,kw
#4	("colorectal cancer"):ti,ab,kw
#5	(colon cancer):ti,ab,kw
#6	(rectal cancer):ti,ab,kw
#7	("esophageal cancer"):ti,ab,kw
#8	("gastric cancer"):ti,ab,kw
#9	("liver cancer"):ti,ab,kw
#10	(hepatocellular carcinoma):ti,ab,kw
#11	("pancreatic cancer"):ti,ab,kw
#12	("cholangiocarcinoma"):ti,ab,kw
#13	(gallbladder carcinoma):ti,ab,kw
#14	#1 OR #2 OR #3 OR #4 OR #5 OR #6 OR #7 OR #8 OR #9 OR #10 OR #11 OR #12 OR #13
#15	MeSH descriptor: [Hematologic Neoplasms] explode all trees
#16	MeSH descriptor: [Multiple Myeloma] explode all trees
#17	MeSH descriptor: [Leukemia] explode all trees
#18	MeSH descriptor: [Lymphoma] explode all trees
#19	("leukemia"):ti,ab,kw
#20	("myeloma"):ti,ab,kw
#21	("lymphoma"):ti,ab,kw
#22	("lymphoproliferative"):ti,ab,kw
#23	("myeloid"):ti,ab,kw
#24	("myelodysplastic"):ti,ab,kw
#25	("lymphoma*"):ti,ab,kw
#26	("Hodgkin"):ti,ab,kw
#27	("Immunoproliferative Small Intestinal"):ti,ab,kw
#28	("AML"):ti
#29	("CLL"):ti
#30	("CML"):ti
#31	("APL"):ti
#32	("HL"):ti
#33	("NHL"):ti
#34	("hematopoietic*"):ti
#35	("haematologic*"):ti

#36	#15 OR #16 OR #17 OR #18 OR #19 OR #20 OR #21 OR #22 OR #23 OR #24 OR #25 OR #26 OR #27 OR #28 OR #29 OR #30 OR #31 OR #32 OR #33 OR #34 OR #35
#37	#14 NOT #36
#38	MeSH descriptor: [Circulating Tumor DNA] explode all trees
#39	("Circulating Tumor DNA"):ti,ab,kw
#40	("ctDNA"):ti,ab,kw
#41	("ct DNA"):ti,ab,kw
#42	("tumor dna circulating"):ti,ab,kw
#43	#38 OR #39 OR #40 OR #41 OR #42
#44	("molecular residual disease*"):ti,ab,kw
#45	("minimal residual"):ti,ab,kw
#46	("MRD"):ti
#47	("residual minimal"):ti,ab,kw
#48	("measurable residual"):ti,ab,kw
#49	#44 OR #45 OR #46 OR #47 OR #48
#50	#43 OR #49
#51	#37 AND #50
#52	#51 AND "2016-2022"

4. Search strategy for clinicaltrial.gov

("Circulating Tumor DNA" OR "ctDNA" OR "ct DNA" OR "molecular residual" OR "minimal residual" OR "MRD" OR "measurable residual" OR "residual minimal")|Studies With Results| ("Neoplasms") NOT ("hematologic neoplasms" OR " myeloma" OR "leukemia" OR "lymphoma" OR "lymphoproliferative" OR "myeloid" OR "myelodysplastic syndrome" OR "Hodgkin" OR "immunoproliferative small intestinal")

5. Search strategy for WHO-ICTRP

Title	Circulating Tumor DNA OR ctdna OR ctDNA OR ct DNA OR circulating tumor DNA OR molecular residual disease OR minimal residual OR minimal residual disease OR minimal residual diseases OR residual minimal disease OR MRD OR measurable residual disease
Condition	cancer OR tumor OR neoplasm OR carcinoma

6. Search strategy for WOS (the web of science)

((((((((((((TS=(Neoplasms)) OR TS=("breast cancer")) OR TS=("lung cancer")) OR TS=("colorectal cancer")) OR TS=("colon cancer")) OR TS=("rectal cancer")) OR TS=("esophageal cancer")) OR TS=("gastric cancer")) OR TS=("liver cancer")) OR TS=(" hepatocellular carcinoma ")) OR TS=("pancreatic cancer")) OR TS=("gallbladder carcinoma ")) OR TS=("cholangiocarcinoma") NOT (((((((((((((((((TS=(Hematologic neoplasms)) OR TS=(Myeloma)) OR TS=(Leukemia)) OR TS=(Lymphoma)) OR TS=("Myelodysplastic Syndromes")) OR AB=(lymphoproliferative)) OR TS=(myeloid)) OR TI=("lymphoma*")) OR TI=("Hodgkin")) OR TS=("Immunoproliferative Small Intestinal")) OR TI=("AML")) OR TI=("CLL")) OR TI=("CML")) OR TI=("APL")) OR TI=("HL")) OR TI=("NHL")) OR TI=("hematopoietic*")) OR TI=("haematologic*"))) AND ((((((((TS=("Circulating Tumor DNA")) OR TS=("ctDNA")) OR TS=("molecular residual")) OR TS=("minimal residual")) OR TS=("MRD")) OR TS=("residual minimal")) OR TS=("measurable residual")) and 论文 or 会议摘要 or 会议录论文 (文献类型) and 2022 or 2021 or 2020 or 2019 or 2018 or 2017 or 2016 (出版年)

Supplementary Table 1 Exposure measurements and outcome information of included studies

Author	ctDNA method	Tumor-inform	Measure time	Outcome	Lead time, median	ROB score
L Cavallone, 2020	ddPCR	Y	During NAT;<br>After NAT and before surgery	RFS<br>OS<br>Recurrence<br>pCR<br>ctDNA rate<br>NPV/PPV-pCR<br>NPV/PPV- recurrence	-	6
YH Chen, 2017	NGS	Y	After surgery	DFS<br>Recurrence<br>ctDNA rate<br>NPV/PPV-recurrence	0.07–8.87 months	6
I Garcia-Murillas, 2019	dPCR	Y	Baseline;<br>After surgery	RFS<br>OS<br>ctDNA rate	10.7 (95% CI: 8.1–19.1) months	8
S Li, 2020	NGS	N	Baseline;<br>After NAT and before surgery	DFS<br>OS<br>Recurrence<br>ORR<br>ctDNA rate<br>ctDNA negative rate	-	8
MJM Magbanua, 2020	PCR-NGS	Y	Baseline;<br>During NAT;<br>After NAT and before surgery	DRFS<br>pCR<br>ctDNA rate<br>ctDNA negative rate<br>NPV/PPV-pCR	-	8
E Ortolan, 2019	ddPCR	Y	After NAT and before surgery;	EFS	-	8

			After surgery	Recurrence<br>ctDNA rate<br>NPV/PPV-recurrence		
M Radovich, 2020	the Foundation-ACT or FoundationOne Liquid assays	N	After surgery	DFS<br>DDFS<br>OS<br>ctDNA rate	-	8
RC Coombes, 2019	PCR-NGS	Y	After surgery	RFS<br>Recurrence<br>ctDNA rate<br>NPV/PPV-recurrence	266 (range 14–721) days or 8.9 months	7
F Riva, 2016	ddPCR	Y	Baseline;<br>After NAT;<br>After surgery	Recurrence<br>pCR<br>ctDNA rate<br>NPV/PPV-pCR	-	7
F Rothe, 2019	ddPCR	Y	Baseline;<br>During NAT;<br>Before surgery	EFS<br>pCR<br>ctDNA rate<br>ctDNA negative rate<br>NPV/PPV-pCR	-	6
H Takahashi, 2016	PCR	Y	Baseline;<br>After surgery	pCR<br>Recurrence<br>ctDNA rate<br>ctDNA negative rate<br>NPV/PPV-pCR	-	7
Y Chen, 2021	PCR	-	Baseline;<br>After NAT and before surgery	OS<br>ORR<br>ctDNA rate<br>ctDNA negative rate	-	7
PH Lin, 2021	NGS	N	Baseline;<br>After surgery	RFS<br>pCR<br>ctDNA rate	-	8

T Yoshinami, 2020	NGS	Y	Before surgery	ctDNA negative rate<br>NPV/PPV-pCR<br>DDFS<br>Recurrence<br>ctDNA rate<br>NPV/PPV-recurrence	-	6
M Lipsyc-Sharf, 2022	NGS/ RaDaR assays	Y	After surgery	Recurrence<br>ctDNA rate<br>NPV/PPV-recurrence	12.4 months	7
Q Zhou, 2022	NGS	Y	Baseline;<br>During NAT;<br>After NAT and before surgery;	pCR<br>ctDNA rate<br>ctDNA negative rate	-	7
SD Cosimo, 2019	dPCR	-	After surgery	Recurrence	20 (range 10–47）months	4
W Janni, 2022	WES and RaDaR assays	Y	After surgery	Recurrence	92 (range 42–308) days	5
E Agostinetto, 2022	WES and Signatera^{TM} assay	Y	Baseline;<br>After NAT and before surgery;<br>After surgery	EFS<br>ctDNA rate	-	5
P Sharma, 2022	NGS	-	After surgery	EFS<br>OS<br>ctDNA rate	-	6
MJM Magbanua, 2021	Signatera	Y	-	pCR	-	7
J Lan, 2022	NGS	N	After surgery	ctDNA rate	-	4
X Zhang, 2019	NGS	N	Before surgery;<br>After surgery	ctDNA rate	-	4
DM Carraro, 2020	-	N	Baseline;<br>During NAT	ctDNA rate	-	4
N Turner, 2022	PCR	Y	After NAT and surveillance	ctDNA rate	-	5
Y Takahashi, 2020	NGS	Y	Baseline	ctDNA rate	-	3
RJ Cutts, 2021	WES and RaDaR assays	Y	After surgery	ctDNA rate	12.89 (range: 3.72–26.04)	4

					months	
F Lynce, 2022	RaDaR assay	Y	Baseline	ctDNA rate	-	4
F Ma, 2018	-	-	Baseline	ctDNA rate	-	3
M Fedyanin, 2020	NGS-ddPCR	Y	After surgery	ctDNA rate	-	4

Note: “-” means not available; ctDNA, circulating tumor DNA; ddPCR, digital droplet polymerase chain reaction; dPCR, digital polymerase chain reaction; DFS, disease-free survival; DRFS, distant disease-free survival; DDFS, distant disease–free survival; EFS, event-free survival; PCR, polymerase chain reaction; NAT, neoadjuvant chemotherapy treatment; NGS, next-generation sequencing; NPV, negative predictive value; PPV, positive predictive value; OS, overall survival; ORR, objective response rate; pCR, pathological complete response; RFS, relapse-free survival; WES, whole-exome sequencing.

Supplementary Table 2 The basic information of the study populations

**Author**	**Histological type, N**	**Grade, N**	**Subtype, N**	**Clinical Stage, N**	**Follow-up, median**
L Cavallone, 2020	Ductal: 25<br>Lobular: 3<br>(Both types: 2)	I: 0<br>II: 5<br>III: 21	TNBC	I: 1<br>II: 19<br>III: 6	63 months post-diagnosis/<br>55 months post-surgery
YH Chen, 2017	-	-	-	-	24 months
I Garcia-Murillas, 2019	IDC: 122<br>ILC: 12<br>Mixed: 5<br>Other: 4<br>Unknown: 1	I: 2<br>II: 45<br>III: 87<br>Unknown:10	ER+ HER2-: 51<br>ER+ HER2+: 29<br>ER- HER2+: 26<br>TNBC: 38	Early	36.3 (range 4.1–73.2) months
S Li, 2020	-	-	HR+/HER2-: 21<br>HR+/HER2+: 8<br>HR-/HER2+: 9<br>TNBC: 6	I: 2<br>II: 23<br>III: 14<br>VI: 5	46 (range 11–68) months
MJM Magbanua, 2020	-	II: 17<br>III: 24<br>Unknown: 19	HR+HER2-：35%<br>HER2-: 23%<br>TNBC: 43%	II/III	4.8 (range 0.5–6.3) years
E Ortolan, 2019	-	II: 1<br>III: 26<br>Unknown: 4	TNBC	II: 24<br>III: 7	3 (range 0.5–6.5) years
M Radovich, 2020	-	I: 1<br>II: 22<br>III: 168<br>Unknown: 5	TNBC	Anatomic<br>I: 45<br>II: 100<br>III: 50<br>Unknown: 1	17.2 (range 0.1–58.3) months
RC Coombes, 2019	-	-	HR+HER2-: 34<br>HR+HER2+: 8<br>TNBC: 7	IA: 1<br>IIA: 1<br>IIB: 14	Up to 4 years

				IIIA: 17<br>IIIB: 2<br>IIIC: 14	
F Riva, 2016	Anaplastic: 1<br>Cli pleomorfic: 1<br>IC-NST: 33<br>Metaplastic: 1	I: 1<br>II: 4<br>III: 31	TNBC	I: 2<br>II: 31<br>III: 3	24 (range 9–36) months
F Rothe, 2019	-	II: 25<br>III: 35<br>Unknown: 9	HR+: 33<br>HR-: 36	-	6.64 (range 0.003–7.94) years
H Takahashi, 2016	-	I/II: 44<br>III: 42<br>Unknown: 1	HER2-: 66<br>HER2+: 21<br>ER/PgR-: 28<br>ER/PgR+: 59	II: 62<br>III: 25	After surgery 23 (range 3–33) months
Y Chen, 2021	-	-	-	-	Up to 24 months
PH Lin, 2021	-	-	ER+HER2-: 41<br>HER2+: 29<br>TNBC: 25	II/III	5.1 years
T Yoshinami, 2020	IDC: 55<br>ILC: 4<br>Others: 3	I/II: 47<br>III: 14	HER2+: 10<br>HER2-: 52<br>ER+: 50<br>ER-: 12	I: 39<br>II: 23	-
M Lipsyc-Sharf, 2022	-	I: 11<br>II: 42<br>III: 30	-	IIA: 2<br>IIB: 24<br>IIIA: 40<br>IIIB: 3<br>IIIC: 14	10.4 (range 6.7–22.8) years from diagnosis and 2 (0–3.9) years from first plasma sample collected on study
Q Zhou, 2022	-	I: 8<br>II: 53<br>III: 80<br>Unknown: 4	HR+: 96<br>TNBC: 46<br>Unknown: 3	-	-
SD Cosimo, 2019	-	-	-	Early	80–200 months

W Janni, 2022	-	-	TNBC: 7<br>HR+/HER2-: 28<br>HER2+: 3	Early	3 years
E Agostinetto, 2022	-	-	HR+/HER2-: 20<br>TNBC: 13<br>HER2+: 11	-	3.30 (range 0.39–5.85) years
P Sharma, 2022	-	-	-	-	3 years
MJM Magbanua, 2021	-	-	HR+/HER2-: 77<br>TNBC: 61	II/III	2.8 years
J Lan, 2022	-	-	HR+/HER2-: 10<br>TNBC: 4<br>HER2+: 6	I: 6<br>II: 8<br>III: 6	-
X Zhang, 2019	Ductal: 93<br>Lobular: 7<br>Mucinous: 1<br>Apocrine: 1	I: 1<br>II: 62<br>III: 30	ER+: 34<br>ER+: 68<br>HER2-: 68<br>HER2+: 27	I: 22<br>II: 62<br>III: 18	At least 5 years or until the end of a patient's life
DM Carraro, 2020	-	-	TNBC	-	
N Turner, 2022	-	-	TNBC	Early	12–24 months
Y Takahashi, 2020	-	-	-	0: 2<br>I: 17<br>II: 12<br>III: 3<br>IV: 4	-
RJ Cutts, 2021	-	-	HR+HER2-: 12<br>HER2+: 7<br>TNBC: 3	Early	24.6 months
F Lynce, 2022	-	-	TNBC	-	12months
F Ma, 2018	-	-	HR-HER2+	Early	-
M Fedyanin, 2020	-	-	HER2+	-	2 years

Note: "-" means not available; ER, estrogen receptor; HR, hormone-receptor; HER2, human epidermal growth factor receptor 2; IC-NST, invasive carcinoma of no special type; IDC, invasive ductal carcinoma; ILC, invasive lobular carcinoma; Mixed, IDC and ILC; N, number; PgR, progesterone receptor; TNBC, triple negative breast cancer.

Supplementary Table 3 ctDNA detection rates at different timepoints

**ctDNA measure timepoint**	**Number of study, N**	**Sample size, N**	I^2	***P* Value**	**Model**	**ctDNA detection rate, %**	**95% CI, %**
Baseline	16	962	88.2%	<0.01	Random	59.40	49.15–69.27
During NAT	5	320	82.3%	<0.01	Random	34.39	18.85–51.77
Before surgery	10	597	94.2%	<0.01	Random	24.27	11.01–40.50
After surgery	16	1092	92.2%	<0.01	Random	23.48	14.59–33.65

Note: ctDNA: circulating tumor DNA; CI: Confidence interval; N, number; NAT: neoadjuvant treatment.

Supplementary Table 4 ctDNA+ negative conversion rate in different periods

ctDNA measure timepoint 1	ctDNA measure timepoints 2	Number of study, N	Sample size, N	I^2	*P* Value	Model	ctDNA+ negative conversion rate, %	95% CI, %
Baseline	During NAT	3	145	38.0%	0.199	Fixed	49.66	41.41–57.92
During NAT	After NAT and before surgery	2	37	94.7%	<0.001	Random	56.44	0.00–100.00
Baseline	After NAT and before surgery	4	180	90.0%	<0.001	Random	72.25	48.68–90.88
Baseline	Before surgery	1	20	-	-	-	30.00	11.89–54.28
Baseline	After surgery	1	60	-	-	-	70.00	56.79–81.15
After NAT and before surgery	After surgery	1	11	--	-	-	90.91	64.99–100.00

Note: “-” means not available; CI, Confidence interval; N, number; NAT, neoadjuvant treatment.

Supplementary Table 5 Risk assessment of bias in cohort studies

Author	Article Type	Selection				Comparability	Outcome			ROB score
		Representativeness of the exposed cohort	Selection of the non-exposed cohort	Ascertainment of exposure	Demonstration that outcome of interest was not present at start of study	Comparability of cohorts on the basis of the design or analysis	Assessment of outcome	Was follow-up long enough for outcomes to occur?	Adequacy of follow up of cohorts	
L Cavallone, 2020	Article	0	1	1	1	0	1	1	1	6
Y.H Chen, 2017	Article	0	1	1	1	1	0	1	1	6
I Garcia-Murillas, 2019	Article	1	1	1	1	1	1	1	1	8
S Li, 2020	Article	1	1	1	1	1	1	1	1	8
M.J.M Magbanua, 2020	Article	1	1	1	1	1	1	1	1	8
E Ortolan, 2019	Article	1	1	1	1	1	1	1	1	8
M Radovich, 2020	Article	1	1	1	1	1	1	1	1	8
R.C Coombes, 2019	Article	1	1	1	1	0	1	1	1	7
F Riva, 2016	Article	1	1	1	1	0	1	1	1	7
F Rothe, 2019	Article	0	1	1	1	0	1	1	1	6
H Takahashi, 2016	Article	1	1	1	1	1	0	1	1	7
Y Chen, 2021	Article	1	1	1	1	0	1	1	1	7
P.H Lin, 2021	Article	1	1	1	1	1	1	1	1	8
T Yoshinami, 2020	Article	1	1	1	1	0	0	1	1	6
M Lipsyc-Sharf, 2022	Article	1	1	1	1	0	1	1	1	7
Q Zhou, 2022	Article	1	1	1	1	0	1	1	1	7

E Agostinetto, 2022	Conference abstract	1	1	0	1	0	0	1	1	5
P Sharma, 2022	Conference abstract	1	1	1	1	0	0	1	1	6
M.J.M Magbanua, 2021	Conference abstract	1	1	1	1	1	0	1	1	7

Note: ROB score, the score by risk assessment of bias.

Supplementary Table 6 Risk assessment of bias in case-control studies

Author	Type	Selection				Comparability	Outcome			ROB score
		Representativeness of the exposed cohort	Selection of the non-exposed cohort	Ascertainment of exposure	Demonstration that outcome of interest was not present at start of study	Comparability of cohorts on the basis of the design or analysis	Assessment of outcome	Was follow-up long enough for outcomes to occur?	Adequacy of follow up of cohorts	
S.D Cosimo, 2019	Conference abstract	0	1	0	0	0	1	1	1	4
W Janni, 2022	Conference abstract	0	1	0	1	0	1	1	1	5

Note: ROB score, the score by risk assessment of bias.

Supplementary Table 7 Risk assessment of bias in case series studies

Author	Type	Risk questions								ROB score
		1. Case series collected in more than one centre, i.e. multi-centre study	2. Is the hypothesis/aim/objective of the study clearly described?	3. Are the inclusion and exclusion criteria (case definition) clearly reported?	4. Is there a clear definition of the outcomes reported?	5. Were data collected prospectively?	6. Is there an explicit statement that patients were recruited consecutively?	7. Are the main findings of the study clearly described?	8. Are outcomes stratified? (e.g., by disease stage, abnormal test results, patient characteristics)	
J Lan, 2022	Article	0	1	0	1	0	0	1	1	4
X Zhang, 2019	Article	0	1	0	0	1	0	1	1	4
D.M Carraro, 2020	Conference abstract	0	1	0	1	0	0	1	1	4
N Turner, 2022	Conference abstract	1	1	1	0	1	0	1	0	5
Y Takahashi, 2020	Conference abstract	0	1	0	0	0	0	1	1	3
R.J Cutts, 2021	Conference abstract	0	1	0	1	0	0	1	1	4
F Lynce, 2022	Conference abstract	0	1	0	1	0	0	1	1	4
F Ma, 2018	Conference abstract	0	1	0	0	0	0	1	1	3
M Fedyanin, 2020	Conference abstract	0	1	0	1	1	0	1	0	4

Note: ROB score, the score by risk assessment of bias.

Supplementary Table 8 Sensitivity analysis of meta-analysis with different outcomes at different times

**Outcome**	**Different timepoints**	**Events**	**HR/RR/OR**	**95% CI**
RSF	Baseline ctDNA	Total	1.95	0.84–4.55
		Omitting E Agostinetto, 2022	2.13	0.78–5.82
		Omiting F Rothe, 2019	2.32	0.87–6.19
		Omitting I Garcia-Murillas, 2019	1.61	0.66–3.91
		Omitting M.J.M Magbanua, 2020	1.80	0.71–4.55
		Omitting P.H Lin, 2021*	2.73	1.20–6.24
		Omitting S.Y Li, 2020	1.47	0.64–3.37
	Before surgery	Total	6.55	2.94–14.61
		Omitting E.Ortolan, 2019	8.15	3.83–17.32
		Omitting E Agostinetto, 2022	5.18	2.64–10.15
		Omitting L Cavallone, 2020	7.49	3.51–16.00
		Omitting M.J.M Magbanua, 2020	5.08	2.44–10.60
		Omitting T Yoshinami, 2020	5.53	2.70–11.33
		Replacing single factor results with the results of multi-factor analysis	6.64	2.46–17.89
	After surgery	Total	6.74	3.73–12.17
		Omitting E Agostinetto, 2022	6.20	3.44–11.17
		Omitting I Garcia-Murillas, 2019	6.13	3.34–11.24
		Omitting I Garcia-Murillas, 2019	6.12	3.26–11.49
		Omitting M Radovich, 2020	8.04	4.40–14.69
		Omitting P.H Lin, 2021	7.80	3.90–15.59
		Omitting P Sharma, 2022	7.71	4.00–14.88
		Omitting R Charles Coombes, 2019	6.17	3.24–11.75

		Omitting Y.H Chen, 2017	6.31	3.35–11.89
		Replacing single factor results with the results of multi-factor analysis	6.51	3.67–11.54
		Removing conference abstracts	7.04	3.63–11.65
OS	After surgery	Total	6.03	1.31–27.78
		Omitting I Garcia-Murillas, 2019	2.80	1.43–5.47
		Omitting M Radovich, 2020	9.60	0.96–95.76
		Omitting P Sharma, 2022*	8.75	0.77–99.70
		Replacing single factor results with the results of multi-factor analysis	7.03	1.77–27.98
Relapse	After surgery	Total	7.11	3.05–16.53
		Omiting E Ortolan, 2019	9.15	3.47–24.13
		Omitting H Takahashi, 2016	6.14	2.69–13.98
		Omiting M Lipsyc-Sharf, 2022	4.89	2.58– 9.25
		Omiting R.C Coombes, 2019	6.52	2.49–17.10
		Omitting S Di Cosimo, 2019	6.62	2.63–16.66
		Omitting W Janni, 2022	8.98	3.18–25.34
		Omiting Y.H Chen, 2017	9.16	3.32–25.31
		Removing conference abstracts	8.61	2.58–28.75
pCR	Baseline ctDNA	Total	0.86	0.38–1.93
		Omitting F Riva, 2016	0.87	0.49–1.56
		Omitting F Rothe, 2019	1.16	0.64–2.11
		Omitting H Takahashi, 2016	0.72	0.39–1.34
		Omitting M.J.M Magbanua, 2020	0.79	0.43–1.45
		Omitting Q Zhou, 2022	0.94	0.46–1.90

Note: ctDNA: circulating tumor DNA; CI: Confidence interval; HR/RR/OR: Hazard ratio/Relative risk/Odds ratio; RFS, relapse-free survival; OS, overall survival; pCR, pathological complete response.
